# Inhibitory Effect of Lithospermic Acid on the HIV-1 Nucleocapsid Protein

**DOI:** 10.3390/molecules25225434

**Published:** 2020-11-20

**Authors:** Mattia Mori, Stefano Ciaco, Yves Mély, Anastasia Karioti

**Affiliations:** 1 Department of Biotechnology, Chemistry and Pharmacy, University of Siena, via Aldo Moro 2, 53100 Siena, Italy; mattia.mori@unisi.it (M.M.); stefano.ciaco@virgilio.it (S.C.); 2 Laboratoire de Bioimagerie et Pathologies, UMR 7021 CNRS, Faculté de Pharmacie, Université de Strasbourg, 74 route du Rhin, 67401 Illkirch, France; yves.mely@unistra.fr; 3 Laboratory of Pharmacognosy, School of Pharmacy, Aristotle University of Thessaloniki, 54124 Thessaloniki, Greece

**Keywords:** lithospermic acid, nucleocapsid protein, catechol, natural products, HIV, molecular modeling, fluorescence-based assays

## Abstract

The HIV-1 nucleocapsid protein (NC) is a desirable target in antiretroviral therapy due to its high conservation among HIV-1 strains, and to its multiple and crucial roles in the HIV-1 replication cycle. Natural products represent a valuable source of NC inhibitors, with the catechol group being a privileged scaffold in NC inhibition. By coupling molecular modeling with NMR spectroscopy and fluorescence-based assays, we disclosed lithospermic acid, a catechol derivative extracted from *Salvia miltiorrhizza*, as a potent and chemically stable non-covalent inhibitor of the NC. Being different from other catechol derivative reported so far, lithospermic acid does not undergo spontaneous oxidation in physiological conditions, thus becoming a profitable starting point for the development of efficient NC inhibitors.

## 1. Introduction

Viral infections represent a global health concern, especially when their spread is rapid and there are no effective drugs or pharmacological strategies to counteract their diffusion and lethality. Recent examples are the current pandemic of coronavirus disease (COVID-19) and the Ebola virus disease [1]. In this scenario, the most longstanding and serious infective virus of the last few decades have undoubtedly been the human immunodeficiency virus 1 (HIV-1), which is the causative agent of the acquired immunodeficiency syndrome (AIDS). HIV/AIDS is still a major public health problem in industrialized and, particularly, in under-developed countries [2]. Most of AIDS patients have access to the combined antiretroviral therapy (cART) which is a combination of drugs acting on multiple targets of the HIV-1 replication cycle such as inhibitors of the reverse transcriptase (RT), protease (PR) and integrase (IN) [3]. Although the cART has notably contributed to decrease the mortality rate of AIDS patients, current drugs are unable to eradicate the virus from the host due to its persistence in cellular or tissue reservoirs [4]. Moreover, the ability of HIV-1 to mutate in response to pharmacological pressure gives rise to drug resistance towards cART drugs [5,6,7], which underlines the need to find novel therapeutic strategies. Among the various approaches undertaken to treat HIV-1 infections, we have long been focused on targeting the HIV-1 nucleocapsid protein (NC), a small zinc-binding protein which is involved in multiple steps of the HIV-1 replication cycle and is highly conserved in several HIV-1 strains [8]. NC exerts a chaperone activity towards nucleic acids thanks to its ability to bind and dissociate rapidly from specific nucleic acid sequences, to destabilize secondary and tertiary structures, and to promote the annealing of complementary sequences [9]. Based on this activity, NC is also thought to play a key role in the reverse transcription process [10,11] and integration [12,13,14]. Targeting the NC by small molecules thus offers the unique opportunity to block the HIV-1 replication cycle at multiple steps with a single agent [8].

In state-of-the-art progress, three classes of NC inhibitors have been explored: (i) covalent modifiers that bind to NC zinc-coordinating residues or directly to the Zn(II) ions that are crucial for NC activity; (ii) small molecules that bind to nucleic acids targets of the NC; (iii) non-covalent NC binders that compete with nucleic acids for the binding site on the NC surface [7]. While NC inhibitors of classes (i) and (ii) are most suited as topical microbicides because of their weak selectivity and specificity for the NC that might lead to cytotoxic effects, class (iii) inhibitors have a higher potentiality for being developed as novel anti-HIV-1 systemic drugs [15,16,17,18,19,20,21,22]. It is worth noting that natural products have played an important role in the discovery of NC inhibitors [16,21,23]. Inspired by natural sources, we have recently characterized the mechanism of action of several catechol and 5,6-dihydroxypyrimidine derivatives as non-covalent inhibitors of the NC endowed with antiretroviral activity in cells infected with wild-type and drug resistant HIV-1 strains [16,24]. Both these scaffolds are highly effective in NC inhibition, although the lead compound nordihydroguaiaretic acid experienced chemical stability and reactivity issues [16].

**Figure 1:**
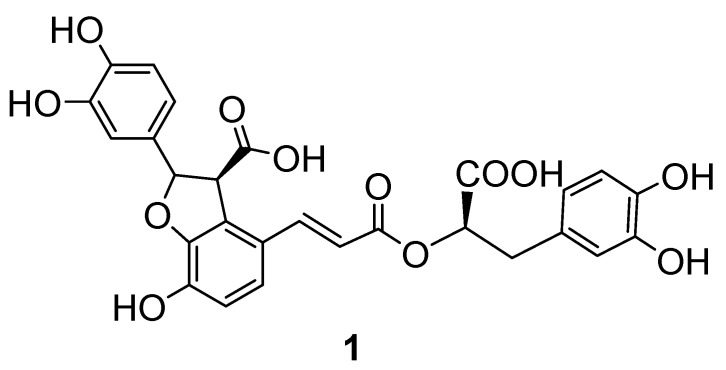
Structure of lithospermic acid isolated from *Salvia miltiorrhiza*.

Natural products are a unique reservoir in the search for new anti-infective agents as they are endowed with structural variety and unprecedented complexity. To date, several studies on the antiviral properties of natural products and plant extracts have revealed many promising compounds [25]. Among the plants studied is *Salvia miltiorrhizza*, commonly known as red sage or Chinese sage [26,27]. The water-soluble extracts of the plant have been shown to exhibit antiviral effects against enterovirus 71, and against HIV-1 integrase activity in vitro and viral replication in vivo [28,29]. Bio-guided isolation revealed lithospermic acid (**1**) (Figure 1) and Salvianolic acid B (**2**) (Figure S9, Supplementary Data) as potent and selective integrase inhibitors, whereas lately Salvianolic acid N, exhibited notable anti-HIV-1 activity and RT and integrase inhibition in vitro [30].

All these compounds are present in the roots of the plant, and from a structural standpoint they are trimers or tetramers of caffeic and phenyllactic acids. In this work, we decided to investigate further the antiviral properties of the main salvianolic acids present in *Salvia miltiorrhizza*, i.e., lithospermic acid (**1**) and Salvianolic acid B (**2**) (Figure 1 and Figure S10). The rational for the selection of these two molecules was the well documented preference of the catechol derivatives to bind to the NC hydrophobic pocket [23]. To further provide insights into the mechanism of action of these natural compounds at a molecular level, the study was performed in two steps: first, molecular modeling was carried out based on a well-established computational protocol to investigate both molecules as candidate non-covalent NC binders; subsequently, the best candidate lithospermic acid was selected for fluorescence studies in order to confirm the NC inhibitory activity. In parallel, nuclear magnetic resonance (NMR) studies were conducted to monitor the chemical stability of the catechol moiety with respect to spontaneous oxidation in experimental conditions.

## 2. Results

### 2.1. Identification of the Two Main Compounds

Lithospermic acid (**1**) was isolated as a brown amorphous powder. In the ESI-MS spectrum (supplementary data) diagnostic fragmentations were observed. A fragment at *m*/*z* = 493.0 [M−CO_2_−H]^−^ was attributed to a neutral loss of CO_2_, whereas the fragment at *m*/*z* = 295 [M−198−CO_2_^−^H]^−^ suggested a further neutral loss of the dihydroxyphenyllactic acid. Finally, fragment *m*/*z* = 185 was assigned to the further loss of the catechol moiety. In the ^13^C NMR/HMBC spectra (Supplementary data), 27 carbons, were observed. Three carbonyl carbons were observed which belonged to one esterified carboxylic carbon at δc 170.4 (C-9) and two free carboxylic carbons at (δc C-9′ 179.3, observed only in the HMBC experiment; C-9′′ 180.5), suggesting that **1** was a phenylpropanoid trimer. Both ^1^H NMR and ^13^C NMR spectra exhibited a plethora of signals in the aromatic area which was in accordance with this assumption. From ^1^H NMR and COSY experiments three aromatic spin systems were identified, two of them as ABX systems (δ_H_ 6.81 brs, H-2′; 6.64 d *J* = 8.2 Hz, H-5′; 6.69, H-6′ and δ_H_ 6.75 brs H-2′′; 6.69, H-5′′; 6.64, H-6′′) and one AB system of two ortho-coupled protons (δ_H_ 6.62 d *J* = 8.3 Hz, H-5; 6.85 d *J* = 8.5 Hz, H-6). The same spectra exhibited signals of a trans olefinic system suggesting the presence of a caffeic acid group (δ_H_ 7.40 d *J* = 15.9 Hz, H-7; 6.04 d *J* = 16.0 Hz, H-8), as well as a –CH(OH)–CH_2_ group consisting of two benzylic protons at δ_H_ 2.91 (dd, *J* = 14.0, 2.6 Hz, H-7′a) and 2.80 (dd, *J* = 14.0, 9.2 Hz, H-7′b) and one oximethine group at δ_H_ 4.79 (dd, *J* = 8.9, 3.6 Hz, H-8′). In the HSQC spectrum these protons corresponded to a methylene carbon and an oxygenated methine carbon at δ_C_ 38.4 and at δ_C_ 75.3, respectively. Finally, a spin system consisting of two methines at δ_H_ 5.63, d, *J* = 5.5 Hz, H-7′′ (δ_C_ 88.8) and δ_H_ 4.05, d, *J* = 5.4 Hz, H-8′′ (δ_C_; 58.4 C-8′′) indicated the presence of a dihydrobenzofuran moiety. In the HMBC spectrum two diagnostic connectivities proved the structure: a common signal between H-8′, H-7, H-8 and C-9 demonstrated the linkage of the dihydroxyphenyllactic moiety to the caffeoyl group similarly as in rosmarinic acid and the common cross-peak between H-7, H-7′′, and H-8′′ of the dihydrobenzofuran group with C-2 proved the linkage of the latter to the aromatic group of the caffeoyl unit. Therefore **1** was assigned to lithospermic acid. Table of its NMR data (Table S1) along with the spectra (Figures S1–S8) are provided in the Supplementary data.

### 2.2. Molecular Modeling

**Figure 2:**
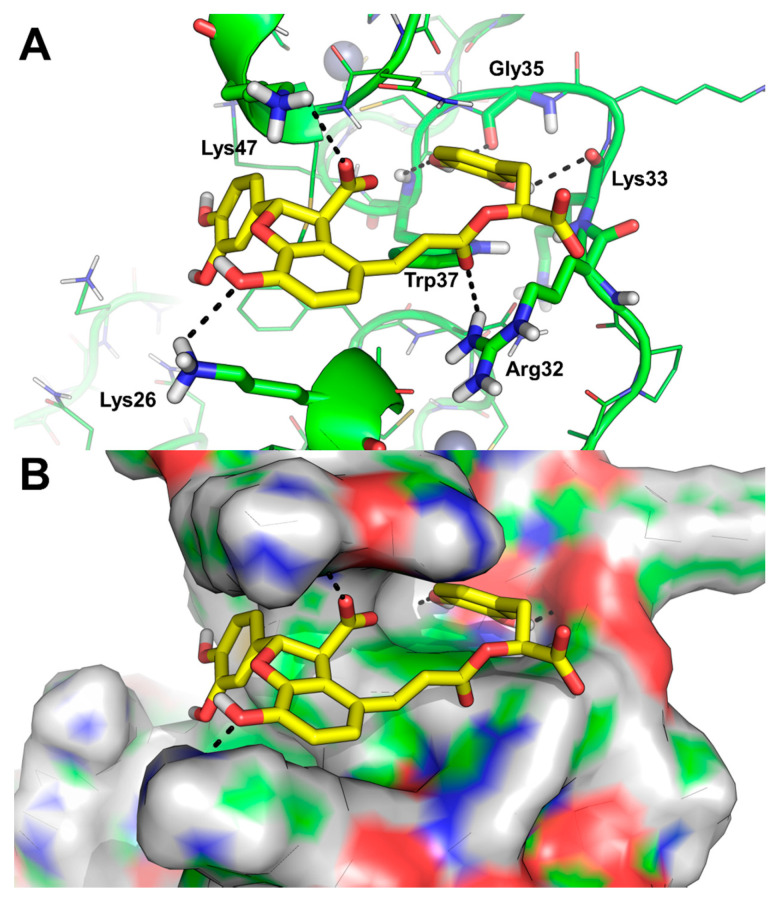
Predicted binding mode of lithospermic acid (**1**) to the NC hydrophobic pocket. (**A**) stick and line representation of the binding mode. The compound is shown as yellow sticks; protein residues are as green line, while the residues H-bonded to the compound are shown as sticks and are labeled; Zn(II) ions are shown as grey spheres. Polar contacts are highlighted by yellow dashed lines. (**B**) Surface representation of the NC binding groove.

The possible binding mode of compounds **1** and **2** was investigated by molecular docking, using a well-established protocol that has been discussed and refined previously [17,19,24,31]. While docking results show that Salvianolic acid B (**2**) is too large for fitting the hydrophobic pocket of the NC in correspondence of Trp37, lithospermic acid (**1**) emerged as a putative NC binder. Indeed, the docking protocol consistently identified a single binding mode of compound **1** in multiple runs (top-ranking 10 poses of each docked ligands were visually inspected, data not shown), showing that the molecule is able to π-π stack over Trp37 side chain, and to establish H-bond interactions with the backbone of Lys33, Gly35, and Trp37 with high similarity to other NC inhibitors and nucleic acid binders [31,32,33,34,35]. In addition, carboxylate ion groups are projected towards the solvent-accessible and highly basic surface of the NC, one of them establishing a polar interaction with the side chain of Lys47 (Figure 2A). The compound is also able to H-bond the side chain of Lys26 and Arg32 (Figure 2A). Analysis of the binding mode further revealed that lithospermic acid (**1**) nicely occupies the basic groove of the NC (Figure 2B) that is generally occupied by single stranded nucleic acid targets of the NC, [32,34,36] which suggests that the compound might inhibit the protein activity by competing with nucleic acids.

### 2.3. NC Inhibition by Lithospermic Acid

Based on the molecular modelling data, only lithospermic acid was chosen and tested for its ability to inhibit the NC-induced destabilization of the cTAR DNA stem-loop, the complementary sequence of the transactivation response element involved in minus strand DNA transfer during reverse transcription [10,11,12]. To this end, we used a well-established fluorescence assay using cTAR DNA labelled with an Alexa488 dye and a Dabcyl quencher at its 5′ and 3′ ends, respectively [37,38]. Destabilization by NC(11-55) of cTAR resulted in an opening of cTAR stem, leading to an increase of Alexa488 fluorescence. Therefore, an inhibitor of NC(11-55) can be detected through its ability to reverse this fluorescence increase.

**Figure 3:**
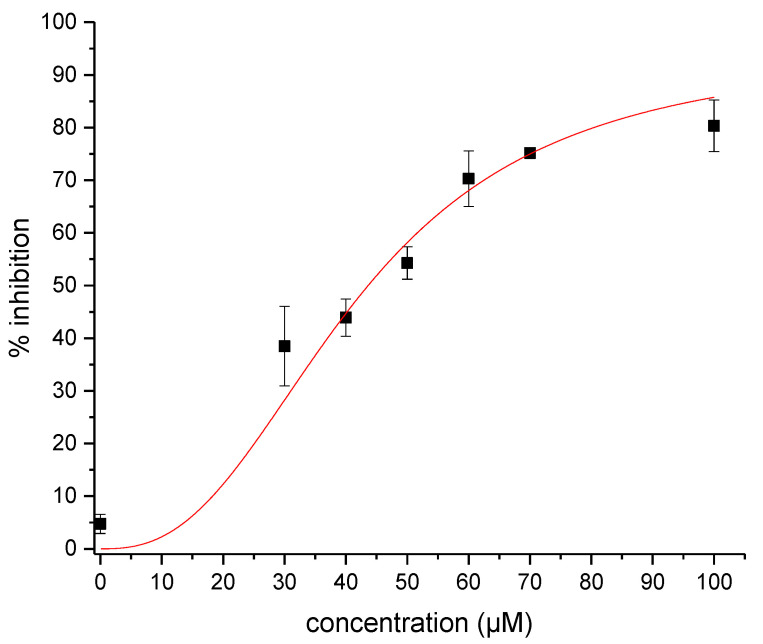
Concentration dependence of the inhibitory activity of lithospermic acid on NC(11-55)-induced destabilization of cTAR. The data points were obtained at 20 °C with 0.1 µM Alexa488-5′-cTAR-3′Dabcyl and 1 µM NC(11-55) in 25 mM TRIS-HCl pH 7.5, 30 mM NaCl and 0.2 mM MgCl_2_. Each point was done in duplicate and corresponds to the mean +/− SEM. The solid line corresponds to the fit to the data points using Equation (2).

Tested at 10 and 100 µM, lithospermic acid (**1**) inhibited the NC-induced destabilization by 5% and 87%, respectively. These percentages did not change with time over one hour, indicating that the inhibition was not time dependent. Next, by monitoring NC inhibition as a function of lithospermic acid concentration (Figure 3), an IC_50_ value of 42 ± 8 µM was found.

### 2.4. Chemical Stability of Lithospermic Acid

Stability by means of NMR revealed that the compounds remained stable under the experimental conditions, as even after one week the NMR signals remained unchanged (Figure S9, Supplementary Data). This chemical behavior is notably different from that observed for the reference NC inhibitor bearing a catechol moiety, i.e., nordihydroguaiaretic acid, which suggests that NC inhibition by **1** is not due to unspecific chemical reactivity. In this respect, **1** could be developed as an effective non-covalent NC inhibitor.

## 3. Discussion

The current pandemic of COVID-19 has highlighted the need to dispose of an arsenal of effective drugs to counteract the spread of viral infections among the population. In this context, HIV/AIDS is still one of the most serious global health threats, with around 1.7 million people infected every year, among which 150,000 are children younger than 15 years old. A preventive vaccine is still not available, while drugs which been developed and are clinically available to around 80% of infected patients, are not able to eradicate the virus from the host [4]. The ability of HIV-1 to mutate its sequence and to replicate in the presence of antiretroviral drugs gives rise to drug-resistance, which underlines the continuous need to develop drugs able to impair the replication cycle of drug-resistant HIV-1 strains. This task is commonly addressed through two main strategies: (i) design of inhibitors of the mutated and drug-resistant form of conventional targets (i.e., RT, IN, and PR); (ii) design of modulators of novel targets, either from the virus or from the host, which are highly conserved among HIV-1 strains or are implicated in the drug resistance mechanisms. One of the most desirable targets of the HIV-1 is the NC, a small and highly basic zinc-binding protein which is involved in multiple steps of the HIV-1 replication cycle thanks to its chaperone activity towards nucleic acids. Despite its unique features as drug target, only a limited number of chemotypes able to inhibit the NC activity in vitro have been developed so far, mostly at the early stage R&D level.

Nordihydroguaiaretic acid is a catechol derivative naturally found in the leaves of *Larrea tridentata*, which has been thoroughly characterized as an antiretroviral agent targeting the HIV-1 NC by computational, biophysical, and virology studies. Unfortunately, the compound proved chemically instable, converting spontaneously into the chemically reactive ortho-quinone form in experimental conditions, and for this reason its development was discontinued. Nevertheless, catechol is a privileged scaffold in NC inhibition, and the identification of chemically stable naturally occurring catechols is a profitable strategy in antiretroviral drug discovery. In this work, we explored the potentiality of two catechol derivatives extracted and purified from *Salvia miltiorrhizza* to behave as NC inhibitors, and to overcome the limitations observed in nordihydroguaiaretic acids. Lithospermic acid (**1**) and Salvianolic acid B (**2**) (Figure 1 and Figure S10) were preliminarily assessed as candidate NC binders by a well-established computational protocol [17,19,24,31]. Docking results highlighted lithospermic acid (**1**) as a candidate non-covalent inhibitor of the NC, whereas the Salvianolic acid B (**2**) proved to be sterically hindered for fitting the NC hydrophobic pocket and not considered further in this study. Lithospermic acid was thus investigated by a well-established fluorescence assay that monitors the inhibition of NC-induced destabilization of specific nucleic acid sequences. Results clearly showed that lithospermic acid is a micromolar inhibitor of the NC, with an IC_50_ value comparable to that of the nordihydroguaiaretic acid, without showing time-dependent NC inhibition. To check whether lithospermic acid inhibits the NC thanks to its catechol group and not because of spontaneous oxidation, chemical stability was monitored in experimental conditions by NMR spectroscopy. NMR results unequivocally showed that the molecule does not undergo spontaneous oxidation, and thus can inhibit the NC without intermolecular cross reactivity. This is indeed a desirable feature of lead-like compounds, which highlights lithospermic acid as a profitable and accessible natural compound inhibitor of the NC for further development. Authors are aware that additional experiments are needed to prove lithospermic acid as a candidate antiretroviral lead; nevertheless, the main finding of this work is the proof that catechol is able to strongly inhibit the NC even in the absence of chemical reactivity subsequent to the oxidation of the catechol to ortho-quinone, as it was observed in the case of nordihydroguaiaretic acid [16]. This evidence also opens the way to the use of chemically stable catechol derivatives in the design of anti-HIV agents.

## 4. Materials and Methods

### 4.1. Isolation, Purification, Characterization and Chemical Stabilitiy of Lithospermic Acid

For the isolation of lithospermic acid and Salvianolic acid B, a *Salvia milthiorriza* enriched fraction was used [39]. This fraction was prepared from a *Salvia miltiorhizza* commercial sample and was kindly provided by the Research Institute of Traditional Chinese Medicine (Tianjin, China) in the framework of a collaboration project between University of Florence and the Tianjin University of Traditional Chinese Medicine) [39]. In brief, dried roots of *Salvia miltiorrhiza* were cut into small pieces and extracted with 50% EtOH. Extracts were concentrated until evaporation of the alcohol and subjected to SFE using macroporous resin D101 as solid phase. The column was washed with excess of acidified water (5% HCl) and consequently with 50% EtOH to afford a purified extract containing 50% Salvianolic acid B (quantification by HPLC UV). 1.0 g of this *Salvia milthiorriza* enriched fraction was subjected to size exclusion column chromatography using Sephadex LH-20 and mixtures of EtOH/H_2_O-MeOH 100% of decreasing polarity as mobile phase. In total, 12 fractions were afforded and were combined by use of HPLC-DAD-MS. In this way 8 sub-groups were obtained (SM-A to SM-H). Fraction C and fraction D, both eluted with EtOH 20%, contained lithospermic acid (**1**). They were re-subjected to Sephadex LH-20 with EtOH 20% to afford pure lithospermic acid (**1**), in total 27.3 mg. As for Salvianolic acid B (**2**) the isolation procedure is described previously [40]. NMR, ESIMS and UV spectra of lithospermic acid (**1**) are available in the Supplementary Material.

The chemical stability of lithospermic acid was monitored by NMR spectroscopy, as described previously [16]. Lithospermic acid was dissolved at 200 μM in 20 mM Hepes (pH 7.5) as buffer and 20 mM Na_2_SO_4_ (for maintaining constant the ionic strength) in D_2_O. Spectra were recorded at 500 MHz at different time points, every 24 h for five consecutive days.

### 4.2. Molecular Modeling

Docking simulations were run according to the protocol described previously [15,16,24]. In brief, the ligand was sketched with VIDA (OpenEye Scientific Software) version 4.4.0.4 [41], while the protonation state was assigned by QUACPAC (OpenEye Scientific Software) version 2.0.0.3 [42]. Ligand energy minimization was carried out by SZYBKI (OpenEye Scientific Software) version 1.10.0.3 using the MMFF94S force field [43]. Conformational analysis was performed with OMEGA (OpenEye Scientific Software) version 3.1.0.3 by storing up to 600 conformers of each molecule [44,45]. Finally, molecular docking was carried out with the FRED program (OpenEye Scientific Solutions) version 3.3.0.3 [46,47]. The NMR structure of the NC in a complex with a small molecule inhibitor (PDB ID: 2M3Z) was used as rigid receptor in molecular docking simulations [35].

### 4.3. Synthesis of NC(11-55) Peptide

The peptide NC(11-55) was synthesized by solid phase peptide synthesis on a 433A synthesizer (ABI, Foster City, CA, USA) as previously described [48,49]. After purification, all fractions containing NC(11-55) were lyophilized and stored at −20 °C. The peptide purity and identity were checked by HPLC and LC-ESI-MS. The zinc-bound form of the peptide was prepared by dissolving the peptide in water, adding a 2.5-fold molar excess of zinc sulphate, and raising the pH to 7.5 by adding concentrated Tris buffer. The peptide concentration was determined by using an extinction coefficient of 5.7 × 10^3^ M^−1^ cm^−1^ at 280 nm.

### 4.4. Oligonucleotides

The doubly labelled cTAR sequence was synthesized, purified, and identified by IBA GmbH Nucleic Acids Product Supply (Göttingen, Germany). Its 5′ terminus was labelled with Alexa488 and the 3′ terminus was labelled with 4-(4′-dimethylaminophenylazo)benzoic acid (Dabcyl). The Alexa488-5′cTAR-3′-Dabcyl sequence was purified by reverse-phase high performance liquid chromatography and polyacrylamide gel electrophoresis. An extinction coefficient at 260 nm of 5.732 × 10^5^ M^−1^.cm^−1^ was used to determine its concentration.

### 4.5. Preparation and Storage of Lithospermic Acid

The main stock of lithospermic acid was solubilized at 20 mM in DMSO and stored at −20 °C. Additional diluted stocks have been prepared at 10, 5, 1, 0.4, and 0.2 mM in DMSO and stored at −20 °C, in order to avoid multiple freeze-thaw cycles of the main stock.

### 4.6. Testing the Inhibitory Activity of the Lithospermic Acid

In the first step, the inhibitory activity of the lithospermic acid was tested at 10 and 100 µM on the NC(11-55)-induced destabilization of cTAR, using 0.1 µM Alexa488-5′-cTAR-3′Dabcyl and 1 µM NC(11-55) in 25 mM TRIS-HCl at pH 7.5, 30 mM NaCl and 0.2 mM MgCl_2_. This buffer was selected as it corresponds to the optimal composition for monitoring the nucleic acid chaperone activity of NC [50] (Lapadat-Tapolsky et al., 1995). The assay was performed at 20 °C in 96-wells black polystyrene CORNING (3686) plates with low-binding surface. Control wells were used with (i) Buffer only, (ii) 0.1 µM Alexa488-5′-cTAR-3′-Dabcyl, and (iii) 0.1 µM Alexa488-5′-cTAR-3′-Dabcyl + 1 µM NC(11-55). The intrinsic fluorescence of the tested compound in buffer was checked in the same plate. The interaction of the compound with the free Alexa488 label was checked as well. The instrument response was controlled by measuring the linear dependence of the fluorescence intensity on the concentration of free Alexa488. Experiments were repeated two times.

The fluorescence intensity and absorbance were measured with a plate reader Xenius (SAFAS, Monaco). For absorbance measurements, we used transparent 96-wells TPP (92096) plates. Fluorescence was recorded in a single point format, with an excitation wavelength of 480 nm and an emission wavelength of 520 nm, slits 10/15 and 10/10, integration time 1 s, 4 measurements in each cell (the average value was taken), full filtering, and PMT voltage of 700 V. In order to check for possible aggregation, the absorbance was measured in a single point format, at 600 nm, with bandwidth 2 nm, integration time 1s, 4 measurements in each cell (the average value was taken).

The percentage of inhibition (%inh) for each concentration of inhibitor (Inh) was calculated using:

(1) $$\%\mathrm{inh}=\frac{I_{\left(\mathrm{cTAR}+\mathrm{NC}\right)}-I_{\left(\mathrm{cTAR}+\mathrm{NC}+\mathrm{inh}\right)}}{I_{\left(\mathrm{cTAR}+\mathrm{NC}\right)}-I_{\left(\mathrm{cTAR}\right)}}\times 100$$

where I_(cTAR)_, I_(cTAR+ NC)_, and I_(cTAR+ NC+ inh)_ correspond to the fluorescence intensity of Alexa488-5′-cTAR-3′-Dabcyl alone, Alexa488-5′-cTAR-3′-Dabcyl in the presence of the NC(11-55) peptide, and Alexa488-5′-cTAR-3′-Dabcyl in the presence of both NC(11-55) and inhibitor, respectively.

### 4.7. IC_50_ Determination

The IC_50_ curve was built by adding increasing concentrations of lithospermic acid to a solution of 0.1 µM Alexa488-5′-cTAR-3′Dabcyl and 1 µM NC(11-55) in 25 mM TRIS-HCl at pH 7.5, 30 mM NaCl and 0.2 mM MgCl_2_. The controls and experimental conditions are the same as in the previous point. Experiments were repeated four times.

The IC_50_ values were obtained by plotting the percentage of inhibition against the inhibitor concentration (C) and fitting it with a modified version of the dose-response equation [51] (Motulsky et al., 1987):

(2) $$\%\;\mathrm{inh}=A_{1}+\frac{\left(A_{2}-A_{1}\right)}{1+10^{\left(\log({\mathrm{IC}}_{50})-\log(C))\times \;p\right)}}$$

where A_1_ and A_2_ represent the percentage of inhibition in the absence and with saturating concentrations of inhibitor, respectively. IC_50_ represents the half maximal inhibitory concentration, and p denotes the Hill coefficient.

## 5. Conclusions

In this work, we identified lithospermic acid, a natural product extracted and purified from *Salvia milthiorriza*, as an inhibitor of the HIV-1 NC protein. Different from catechol derivatives previously identified as NC inhibitors [16,23], lithospermic acid provides a strong inhibition of the NC without undergoing spontaneous oxidation. Molecular modeling illustrated that lithospermic acid might fit the hydrophobic pocket of the NC, possibly competing with nucleic acids targets of this protein within the HIV-1 replication cycle. Overall, lithospermic acid emerged as a profitable and chemically stable catechol derivative able to inhibit the NC, which is worthy of further investigations and development.

## Supplementary Materials

The following are available online. Figure S1: Structure of lithospermic acid, Figure S2: ^1^H NMR spectrum of lithospermic acid (D_2_O, 400 MHz), Figure S3: ^13^C NMR spectrum of lithospermic acid (D_2_O, 100.3 MHz), Figure S4: COSY spectrum of lithospermic acid (D_2_O, 400 MHz), Figure S5: HSQC spectrum of lithospermic acid (D_2_O, 400 MHz), Figure S6: HMBC spectrum of lithospermic acid (D_2_O, 400 MHz) Figure S7: ESI-MS spectrum of lithospermic acid, Figure S8: HPLC-PDA spectrum of lithospermic acid, Figure S9: ^1^H-NMR spectra conducted on executive days to confirm the stability of lithospermic acid (1), Figure S10: Structure of Salvianolic acid B, Table S1: ^1^H and ^13^C NMR data of lithospermic acid (D_2_O, 400 MHz).
