# Supplementary material for: Inhibitory Effect of Lithospermic Acid on the HIV-1 Nucleocapsid Protein
Source: Molecules. 2020 Nov 20;25(22):5434. doi: 10.3390/molecules25225434 (PMC7699738; doi:10.3390/molecules25225434)
Supplement: Supplementary file 1 [file molecules-25-05434-s001.pdf]

molecules

MDPI

Communication

Inhibitory Effect of Lithospermic Acid on the HIV-1 Nucleocapsid Protein

Mattia Mori [1,†], Stefano Ciaco [1,2,†], Yves Mély [2] and Anastasia Karioti [3,*]

[1] Department of Biotechnology, Chemistry and Pharmacy, "Department of Excellence 2018-2022", University of Siena, via Aldo Moro 2, 53100 Siena, Italy; mattia.mori@unisi.it (M.M.); stefano.ciaco@virgilio.it (S.C.)
[2] Laboratoire de Bioimagerie et Pathologies, UMR 7021 CNRS, Université de Strasbourg, Faculté de Pharmacie, 74 route du Rhin, 67401 Illkirch, France; yves.mely@unistra.fr
[3] Laboratory of Pharmacognosy, School of Pharmacy, Aristotle University of Thessaloniki, University Campus, 54124 Thessaloniki, Greece
* Correspondence: akarioti@pharm.auth.gr; Tel.: +30-2310-990356
† These authors contributed equally to this work.

Supplementary Data

Index

FigureS1; Structure of lithospermic acid 2
Table S1: ^{1}H and ^{13}C NMR data of lithospermic acid (D_2O, 400 MHz) 2
Figure S2: ^{1}H NMR spectrum of lithospermic acid (D_2O, 400 MHz) 3
Figure S3: ^{13}C NMR spectrum of lithospermic acid (D_2O, 100.3 MHz) 3
Figure S4: COSY spectrum of lithospermic acid (D_2O, 400 MHz) 4
FigureS5: HSQC spectrum of lithospermic acid (D_2O, 400 MHz) 4
FigureS6: HMBC spectrum of lithospermic acid (D_2O, 400 MHz) 5
Figure S7: ESI-MS spectrum of lithospermic acid 6
Figure S8: HPLC-PDA spectrum of lithospermic acid 6
Figure S9: ^{1}H-NMR spectra conducted on executive days to confirm the stability of lithospermic acid (**1**) 7
Figure S10: Structure of Salvianolic acid B 8

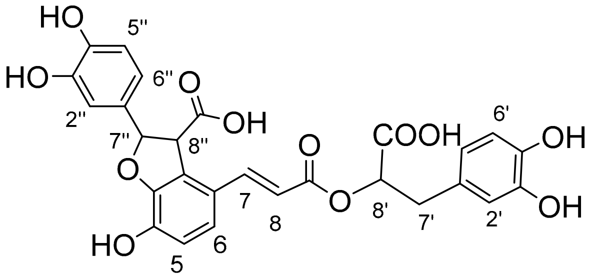

Figure S1. Structure of lithospermic acid.

Table S1. ^{1}H and ^{13}C NMR data of lithospermic acid (D_2O, 400 MHz).

Assignment	δ_H	δ_C
1	-	123.0
2	-	130.4
3	-	148.5
4	-	144.3
5	6.62, d, (8.3 Hz)	118.6
6	6.85, brd, (8.5 Hz)	123.1
7	7.40, d, (15.9 Hz)	144.1
8	6.04, d, (16.0 Hz)	117.5
9	-	170.4
1′	-	132.1
2′	6.81, brs	119.2
3′	-	145.7
4′	-	144.7
5′	6.64, d, (8.2 Hz)	117.9*
6′	6.59 os	119.9
7′	2.91, dd, (14.0,2.6 Hz)<br>2.80, dd, (14.0, 9.2 Hz)	38.4
8′	4.79, d, (8.9, 3.0 Hz)	75.3*
9′	-	179.3
1′′	-	135.1
2′′	6.75, brs	115.0
3′′	-	146.0
4′′	-	145.9
5′′	6.69, os	117.9*
6′′	6.64, os	119.8
7′′	5.63, d, (5.5 Hz)	88.8
8′′	4.05, d, (5.4 Hz)	58.4
9′′	-	180.5

*Values might be interchanged; os.: overlapped signals; proton signals deduced with the help. Some carbon signals were assigned by HSQC and HMBC experiments

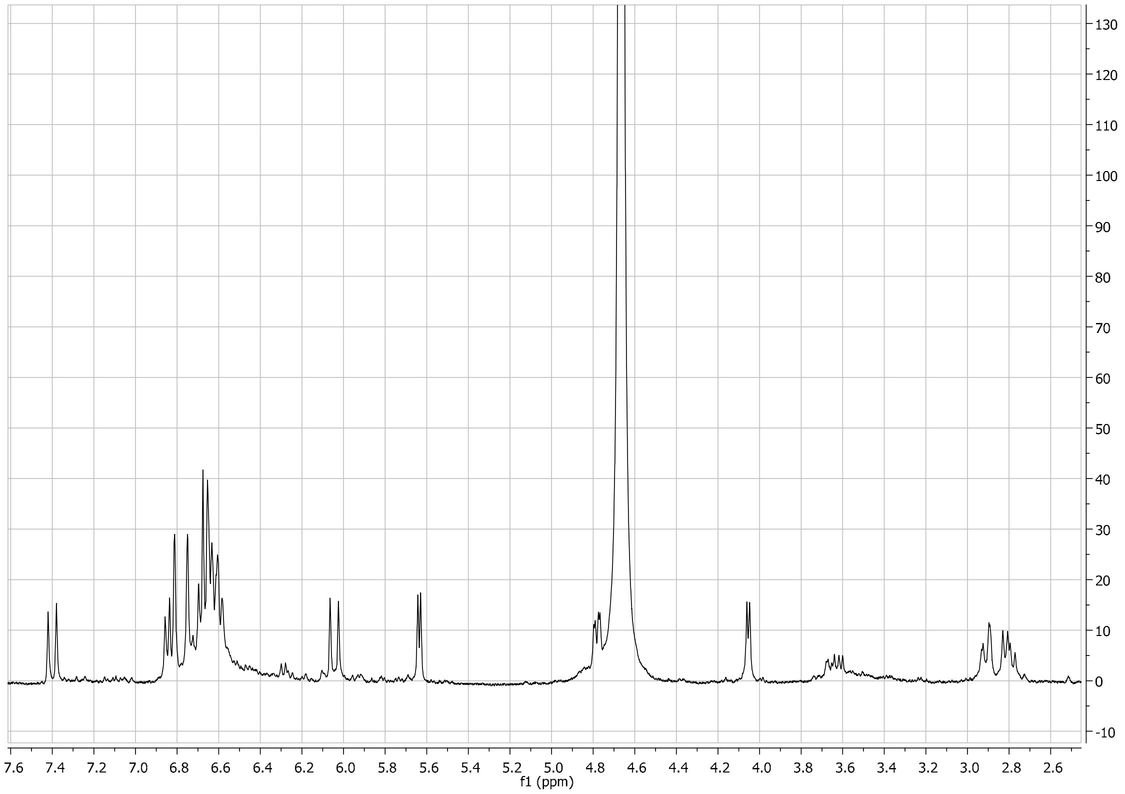

Figure S2. ^1H NMR spectrum of lithospermic acid (D_2O, 400 MHz).

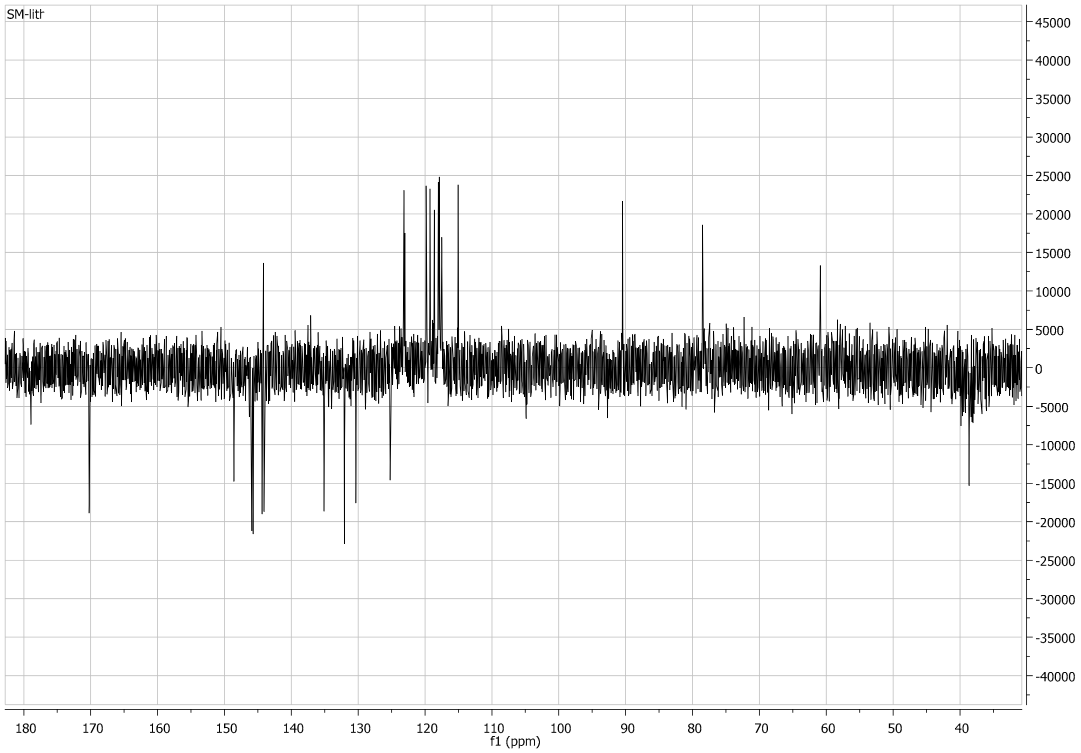

Figure S3. ^{13}C NMR spectrum of lithospermic acid (D_2O, 100.3 MHz).

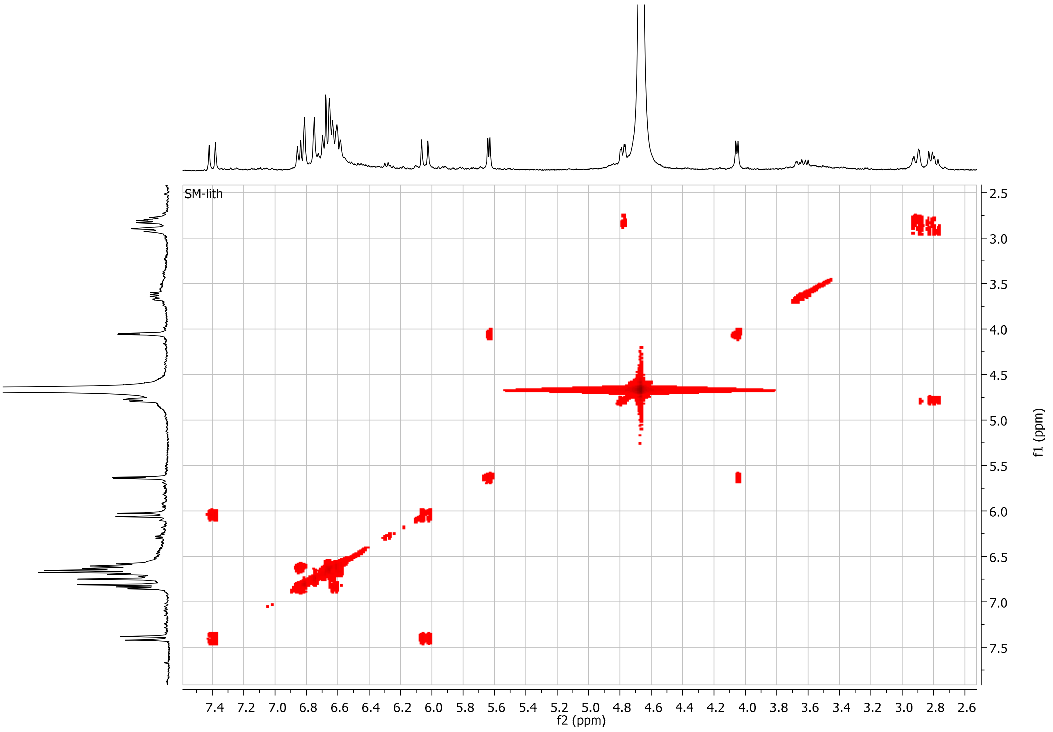

Figure S4. COSY spectrum of lithospermic acid (D_2O, 400 MHz).

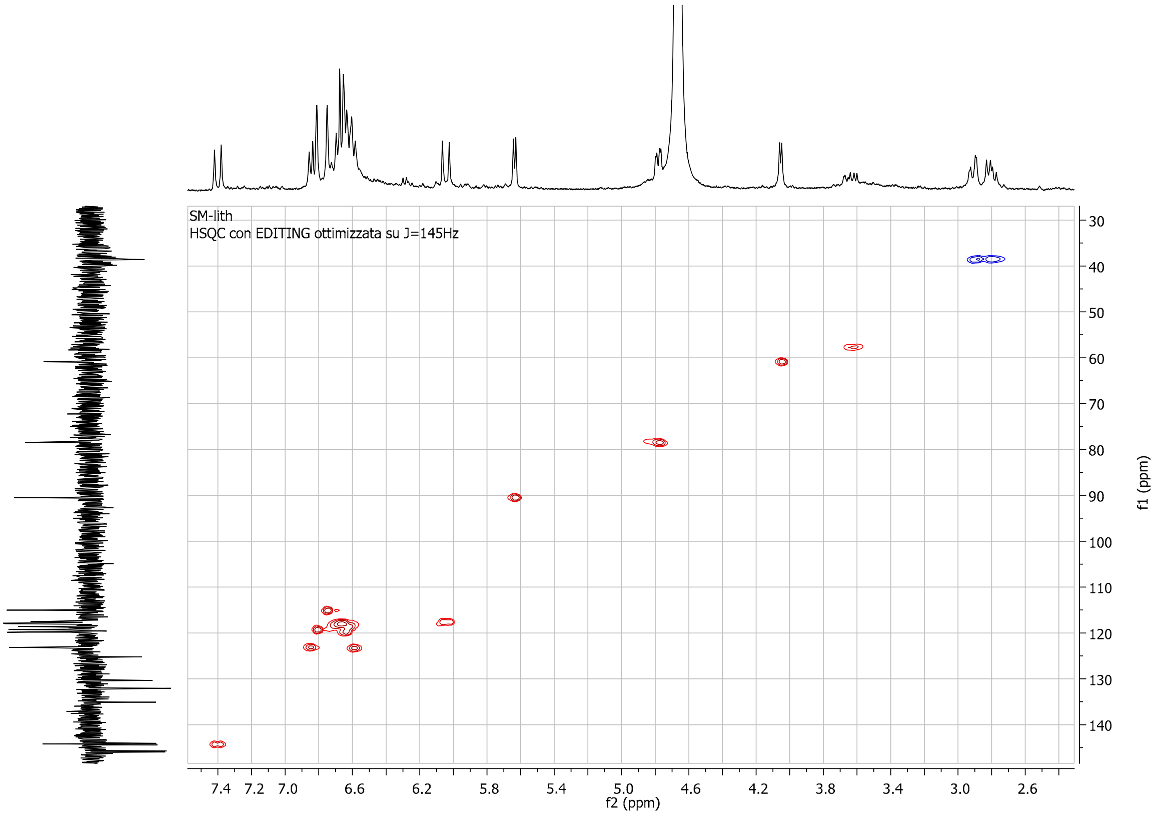

Figure S5. HSQC spectrum of lithospermic acid (D_2O, 400 MHz).

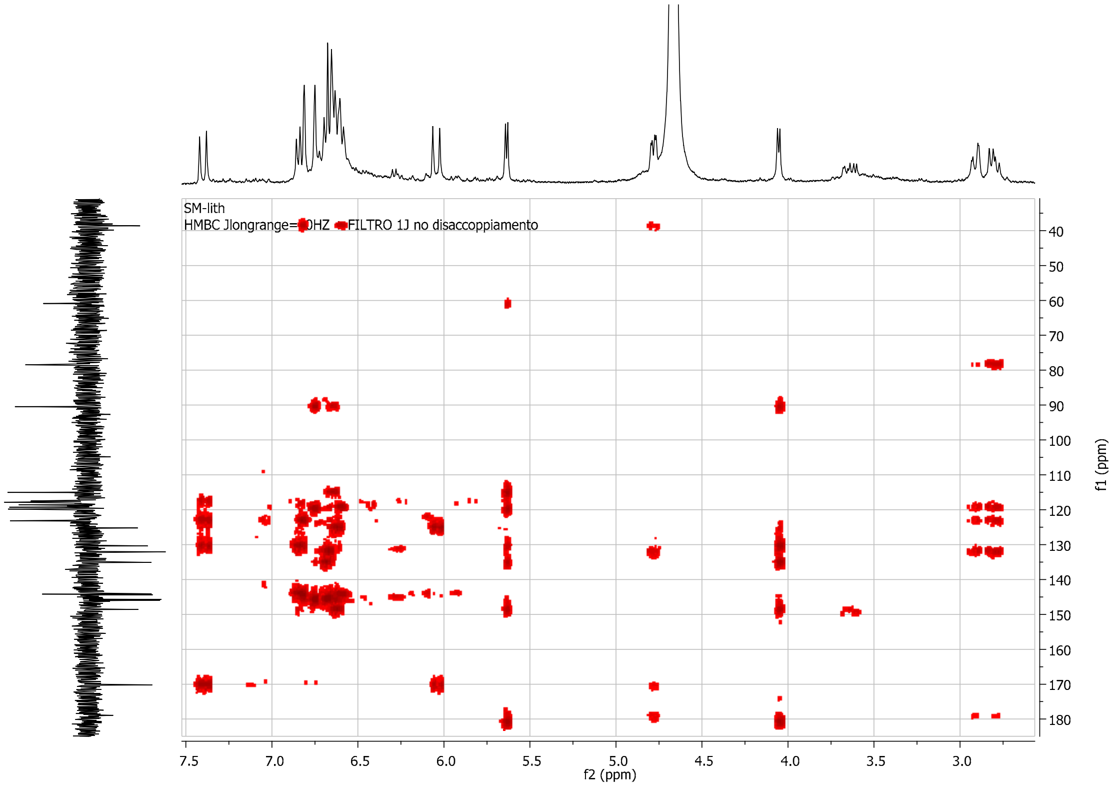

Figure S6. HMBC spectrum of lithospermic acid (D_2O, 400 MHz).

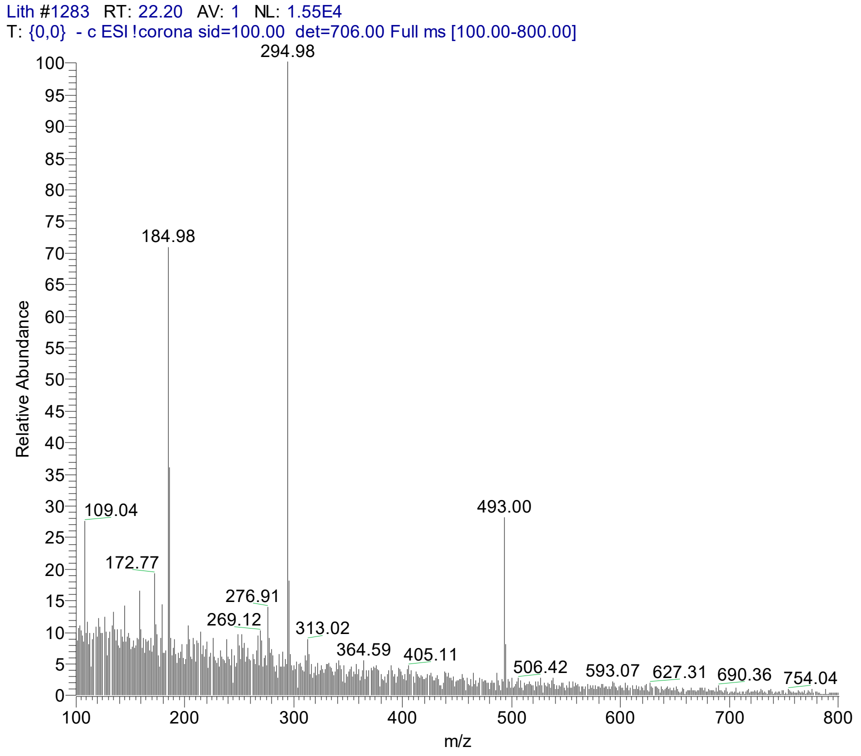

Figure S7. ESI-MS spectrum of lithospermic acid.

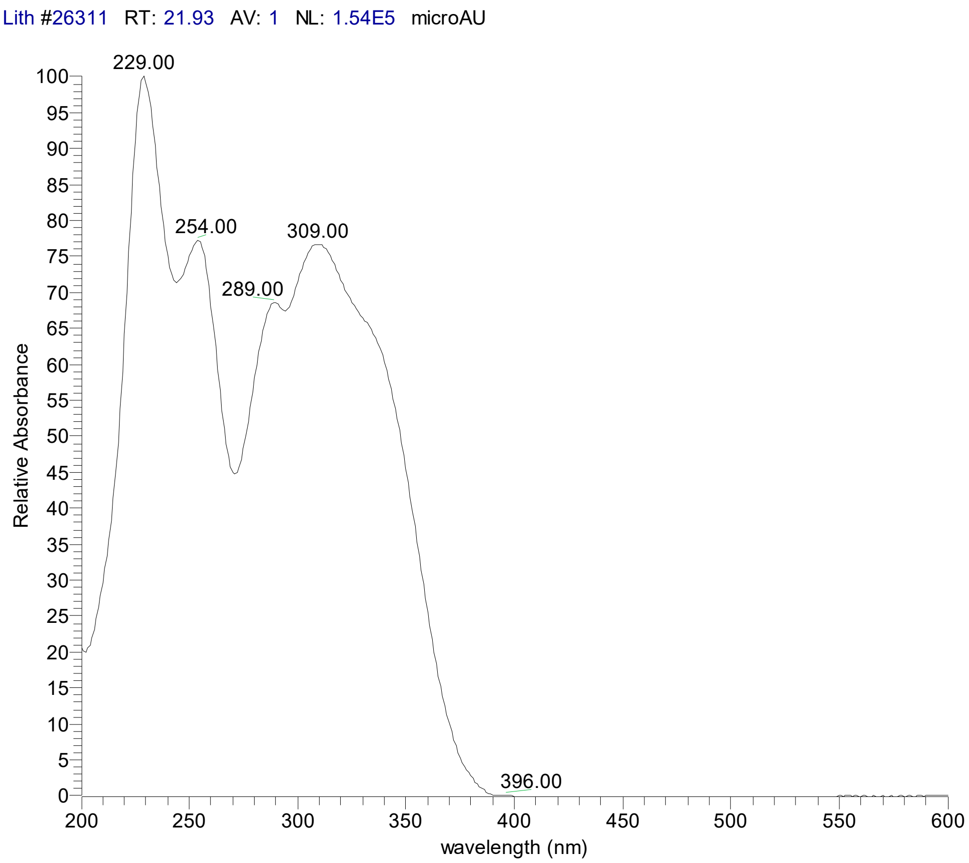

Figure S8. HPLC-PDA spectrum of lithospermic acid (254, 289, 309, 334 nm).

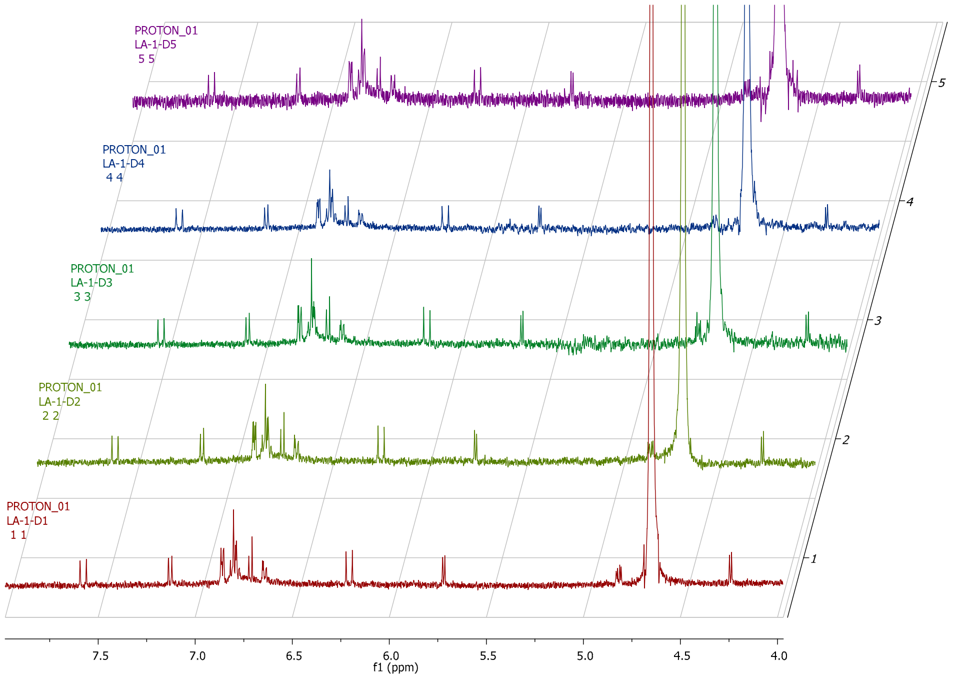

Figure S9. ^{1}H-NMR spectra conducted on executive days to confirm the stability of lithospermic acid (**1**).

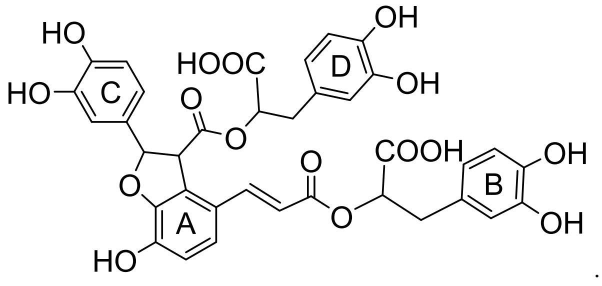

Figure S10. Structure of Salvianolic acid B (virtually screened) given for comparison reasons.
